# A Comparative Study of an Anti-Thrombotic Small-Diameter Vascular Graft with Commercially Available e-PTFE Graft in a Porcine Carotid Model

**DOI:** 10.1007/s13770-021-00422-4

**Published:** 2022-02-15

**Authors:** Kyo Seon Lee, Mukhammad Kayumov, Gladys A. Emechebe, Do-Wan Kim, Hwa-Jin Cho, Yun-Jin Jeong, Dong-Weon Lee, Jun-Kyu Park, Chan-Hee Park, Cheol-Sang Kim, Francis O. Obiweluozor, In-Seok Jeong

**Affiliations:** 1 Department of Thoracic and Cardiovascular Surgery, Chonnam National University Hospital and Medical School, 160 Baekseo-ro, Dong-gu, Gwangju, 61469 Republic of Korea (South Korea); 2 Department of Pediatrics, Chonnam National University Children’s Hospital and Medical School, Gwangju, 61469 Republic of Korea; 3 School of Mechanical Engineering, Chonnam National University, Gwangju, 61469 Republic of Korea; 4 CGBio Co. Ltd., 244 Galmachi-ro, Jungwon-u, Seongnam, 13211 Republic of Korea; 5 Department of Mechanical Engineering Graduate School, Chonbuk National University, 567 Baekje-daero, Deokjin-gu, Jeonju, 54896 Republic of Korea; 6 Department of Bionanosystem Engineering Graduate School, Chonbuk National University, Jeonju, 54896 Republic of Korea

**Keywords:** Electrospinning, 3D printing, Nanofiber, Vascular graft, Endothelialization

## Abstract

**Background:**

We have designed a reinforced drug-loaded vascular graft composed of polycaprolactone (PCL) and polydioxanone (PDO) via a combination of electrospinning/3D printing approaches. To evaluate its potential for clinical application, we compared the *in vivo* blood compatibility and performance of PCL/PDO + 10%DY grafts doped with an antithrombotic drug (dipyridamole) with a commercial expanded polytetrafluoroethylene (*e*-PTFE) graft in a porcine model.

**Methods:**

A total of 10 pigs (weight: 25–35 kg) were used in this study. We made a new 5-mm graft with PCL/PDO composite nanofiber via the electrospinning technique. We simultaneously implanted a commercially available *e*-PTFE graft (*n* = 5) and our PCL/PDO + 10%DY graft (*n* = 5) into the carotid arteries of the pigs. No anticoagulant/antiplatelet agent was administered during the follow-up period, and ultrasonography was performed weekly to confirm the patency of the two grafts *in vivo*. Four weeks later, we explanted and compared the performance of the two grafts by histological analysis and scanning electron microscopy (SEM).

**Results:**

No complications, such as sweating on the graft or significant bleeding from the needle hole site, were seen in the PCL/PDO + 10%DY graft immediately after implantation. Serial ultrasonographic examination and immunohistochemical analysis demonstrated that PCL/PDO + 10%DY grafts showed normal physiological blood flow and minimal lumen reduction, and pulsed synchronously with the native artery at 4 weeks after implantation. However, all *e*-PTFE grafts occluded within the study period. The luminal surface of the PCL/PDO + 10%DY graft in the transitional zone was fully covered with endothelial cells as observed by SEM.

**Conclusion:**

The PCL/PDO + 10%DY graft was well tolerated, and no adverse tissue reaction was observed in porcine carotid models during the short-term follow-up. Colonization of the graft by host endothelial and smooth muscle cells coupled with substantial extracellular matrix production marked the regenerative capability. Thus, this material may be an ideal substitute for vascular reconstruction and bypass surgeries. Long-term observations will be necessary to determine the anti-thrombotic and remodeling potential of this device.

**Supplementary Information:**

The online version contains supplementary material available at 10.1007/s13770-021-00422-4.

## Introduction

Vascular atherosclerosis is among the leading causes of numerous diseases, which in most cases result in death [1]. Vascular graft surgery has been regarded as an alternative treatment option for severe diseases, such as cardiovascular atherosclerosis. Traditionally, autologous vessels were the gold standard. However, in the absence of an appropriate autologous vessel graft, diseased vessel replacement is often reliant on a suitable synthetic prosthesis. Commercially available large-caliber vascular grafts made of expanded polytetrafluoroethylene (*e*-PTFE) and polyethylene terephthalate (PET) have been used successfully in the clinic [2–5]. However, the application of these conduits for blood vessel replacement in the coronary arteries and lower extremities, where the diameters are < 6 mm, is often challenging, and they eventually fail. This has led to a great deal of bioengineering research to develop efficient alternatives [6].

Synthetic vascular grafts have been studied in small-diameter applications for more than three decades. However, most failed or were not satisfactory for clinical use. On the other hand, a cell-based approach has been used to generate a tissue-engineered scaffold with an abundant extracellular matrix (ECM). Regardless of the long-term maturation phase of this graft *ex vivo* [7], significant success has been obtained in large animal models [8–10]. However, numerous challenges, such as quality assurance, logistics, and cost, place limits on the clinical translation of cell-based approaches. Bioengineered blood vessels produced via a decellularization approach have potential in terms of low immunogenicity and enhanced translation. The results of the phase II human trial demonstrated the feasibility of this approach, but further studies are needed to confirm its efficacy [11]. Similarly, traditional synthetic prosthetic conduits, such as those made from *e*-PTFE and PET, failed to improve clinical outcomes in small-diameter vessels due to clotting, infection, pseudoaneurysm, thrombosis, occlusion from myointimal hyperplasia, congenital heart failure due to zero somatic growth, kinking, the need for pre-clotting, seroma formation, and venous hypertension [12].

The introduction of nanotechnology for bioengineering vascular conduits is expected to deliver the “holy grail” of regenerative medicine, i.e., the ability to engineer a scaffold that can mimic the nanostructure of the native vasculature in terms of both mechanical and biological performance. Electrospinning of natural materials and synthetic polymers into nanofibers is a typical application of nanotechnology for tissue engineering [13–16]. This approach has numerous advantages, including high porosity, high surface-area-to-volume ratio, and ease of incorporation of cell signaling molecules, thus mimicking the dimensions and structures of native collagen and elastin [17–19]. He W et al. used electrospinning to fabricate a fibrous scaffold made of poly(l-lactic acid)-co-poly(e-caprolactone) composite, and the results showed enhanced endothelial attachment and spreading *in vitro* [20]. However, *in vitro* data alone cannot be relied upon for better *in vivo* performance of engineered vascular graft due to the dynamic nature of the blood system. Previous *in vivo* studies utilizing biodegradable polymers such as poly(glycerol sebacate) [21], polylactide [22], polycaprolactone [23, 24], and polyurethane [25] as small-diameter vascular graft induced thrombosis and intimal hyperplasia(IH), which are the major barrier towards positive translation. Thus, rapid endothelization of the graft lumen after implantation holds the key to long-term patency. Hence, recent studies are now focused on surface modification [26, 27], nitric oxide release [28, 29], and antithrombotic drug incorporation [30, 31] in the vascular prosthesis in other to prevent thrombosis, IH and at the same time promote rapid endothelialization. Among these strategies drug release approach seems to be a quick and easy way to enhance the biofunctions of biodegradable grafts. In an *in vivo* study, antiproliferative drug such as paclitaxel has been exploited when loaded in polycaprolactone vascular prosthesis to achieve high patency and inhibit smooth muscle proliferation [31]. However, this drug failed to meet other requirement such as rapid endothelialization due to its anti-proliferative property towards endothelial cells.

Dipyridamole is an anti-thrombogenic drug is among the most commonly prescribed drugs in the United States [32] after approval by the FDA. The primary mechanism of action was its ability to inhibit smooth muscle proliferation via suppression of adenosine uptake and cyclic nucleotide phosphodiesterase in cells which subsequently increased intracellular cAMP and cGMP levels [33, 34]. In another study, dipyridamole has been found to promote vascular endothelial cells proliferation [35] thus rendering it as a solution that will overcome the limitation of current strategies.

In our previous study [36], we fabricated a structurally reinforced biodegradable vascular conduit (< 6 mm) by the electrospinning method, coupled with in-depth *in vitro* and mechanical characterization. These grafts had several advantages, including excellent tensile properties, good *in vitro* biocompatibility with human endothelial cells (ECs), and favorable antithrombotic properties. However, immunofluorescence staining and a comparative study with commercially available vascular prosthesis was part of the study limitations. In this study, we compared the *in vivo* blood compatibility and performance of polycaprolactone and polydioxanone (PCL/PDO) grafts doped with an antithrombotic drug (dipyridamole) with a commercial graft (*e*-PTFE), for up to 4 weeks in a porcine model. Here, we evaluated the potential of these grafts for clinical application.

## Materials and methods

Materials used for this study are poly-ε-caprolactone (PCL) (Mn 80,000) and PDO (Resomer® X 206 S; CAS 29223-92-5) which were purchased from Sigma-Aldrich (St. Louis, MO, USA) and used without modification. 1,1,1,3,3,3-Hexafluoro-2-propanol (HFIP) and dipyridamole were purchased from Tokyo Chemical Industry Co., Ltd. (Tokyo, Japan). Medical grade biodegradable PCL granules were purchased from ROKIT (Seoul, South Korea).

**Fig. 1:**
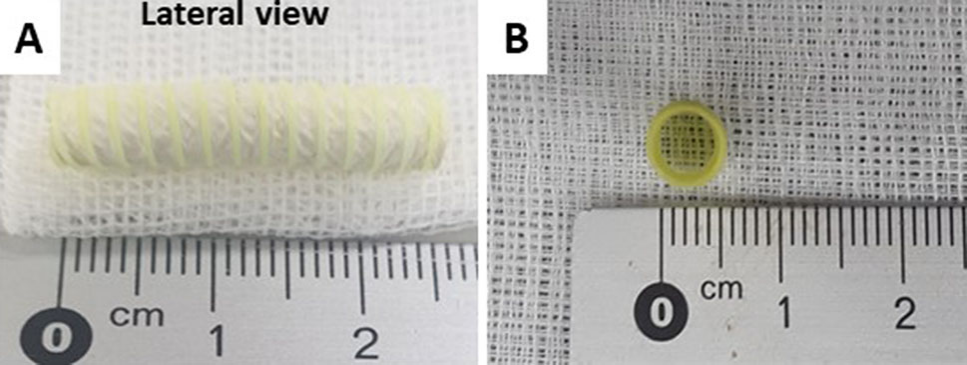
Photo shows an electrospun PCL/PDO vascular graft with an inner diameter of ~ 5 mm. **A** Lateral view **B** cross-section view

We utilized a dual electrospinning approach to fabricate a nano-fibrous PCL/PDO vascular graft with an inner diameter of 5 mm similar to our previous report [36] with minor modification (Fig. 1). Briefly, 0.3 g of PCL was dissolved in a vial containing 3 mL of chloroform and ethyl alcohol mixture (5:1 volume ratio), and in a separate vial, 0.285 g of PDO pellets were placed in 3.56 mL HFIP (8% w/v). For the mat containing dipyridamole (DY) at 5,7, and 10% (w/w) of the drug, 0.0146, 0.02, and 0.0293 g of DY respectively was added to a vial containing either PCL or PDO solution. The polymer solutions were poured into a separate (12 ml) syringe connected to a flexible silicon tubing that was fitted to a programmable pump adjusted to a 1 ml/h flow rate through a 21G needle for both solutions. A high voltage generator ≈ 16.1 kV was applied to both solutions at a gap distance of 80 mm. First, the luminal part of the graft was fabricated by spinning only the PDO solution to the desired thickness (≈ 15 min depending on the mandrel length). Then, simultaneously, both solutions (PCL and PDO) were electrospun to the desired thickness as the spinneret moved in a transverse longitudinal direction. Depending on the mandrel size, different IDs can be fabricated. Hence, for (≈ 5 mm ID) vascular graft, 5 mm steel mandrel rotating at 1700 RPM was used until the desired thickness, based on measurement with a Vernier caliper was obtained. Subsequently, we attached a PCL (160 µm) reinforcement on the outer surface of the graft via a 3D printing system. The printing apparatus consists of a custom 3D bio-printing equipped with a three-axis x-y-z translation stage, dispenser, nozzle, compression/heat controller, and software system. Bio-PCL pellets were melted at 90 °C in a heating dispenser for 30 min, and the PCL strands were deposited via a 160 µm nozzle when a pressure of around 400 to 450 kPa was applied. As the printing head moves slowly in the horizontal axis (3 µm from the mandrel), the mandrel rotates clockwise. The fabricated grafts were cut to the desired length and sterilized with ethylene oxide and placed in a vacuum oven (45 °C) for 48 h to remove residual HFIP, chloroform, and ethyl alcohol. The samples were stored at room temperature before being used.

### Drug entrapment efficiency

We evaluated the drug entrapment efficiency of the nanofiber samples after vacuum drying for 48 h at 40 °C. We prepared a nanofiber mat of known area (1X1 cm) and transferred it into 1 ml of HFIP. The amount of drug in the solutions was calculated by UV analysis and compared with the initial amount of drug-loaded during the electrospinning process.

$${\text{Entrapment}}\,{\text{efficiency}}\left( \% \right) = \frac{{{\text{Mass}}\,{\text{of}}\,{\text{maximum drug}}\,{\text{released}}}}{{{\text{Mass of}}\,{\text{total}}\,{\text{drug}}\,{\text{added}}}} \times 100$$

### Drug release study

Drug release from PCL/PDO nanofibers loaded with three different concentrations of DY 5%, 7%, and 10% (w/w) with respect to the total polymer weight was analyzed separately (*n* = 3 each). The polymer concentration remained 10% and 8%(w/v) for PCL and PDO respectively. The nanofiber scaffold containing different drug concentrations 5, 7, and 10%(w/w) DY were named PCL/PDO + 5%DY, PCL/PDO + 7%DY, and PCL/PDO + 10%DY respectively. Pre-weighted nanofibers (≈ 0.5 g) were placed in 5 mL of phosphate-buffered saline (PBS) (pH 7.4) at 37 °C with mild shaking. At designated time points, 1 mL of release medium was pipetted out and kept at − 20 °C, and the volume was replaced with an equal amount of fresh PBS. The DY concentration in the release medium was measured by UV–vis spectroscopy (SCINCO Mega-800; Scinco Co. Ltd., Seoul, South Korea) at a wavelength of 405 nm. A standard curve was plotted using a series of known dipyridamole contents in 50% ethanol/PBS.

### Platelet adhesion test

A platelet adhesion test was conducted similar to a previous report [37]. Blood was drawn from the ulnar vein of a healthy volunteer, without any medication, and transferred into a tube containing ethylenediaminetetraacetic acid (EDTA) (to prevent any further blood activation) that was mixed in a ratio of 9:1. The treated blood was centrifuged at 2500 rpm for 5 min to separate red blood cells and platelet-rich plasma (PRP). PRP was transferred to a separate tube. Sterile samples of PCL/PDO and PCL/PDO + 10%DY nanofiber measuring (1X1cm each) X3 were incubated with 100 ml of PRP at 37 °C and 5% CO_2_/95% air for 20 min. The samples were then washed three times with PBS which was subsequently fixed by 0.1% glutaraldehyde solution. Finally, after drying at room temperature, the morphology of the samples was assessed by SEM. Triplicate samples were used for this study and PCL/PDO nanofiber mat was used as a negative control.

### *In vivo* animal study

All animal studies were performed following the ethical guidelines of Chonnam National University Hospital and Medical School, South Korea. All protocols were approved by the Animal Ethics Committee(CNUHIACUC-21044). Five castrated adult pigs (weight: 25–35 kg) were purchased from a local pig farm. Each pig was caged separately and maintained at the Animal Center of Chonnam National University Hospital and Medical School, Gwangju, South Korea. The pigs were fed with standard food and the cages were cleaned and disinfected daily.

Each pig was implanted with either PCL/PDO + 10%DY or *e*-PTFE grafts measuring 2 cm in length on the left and right carotid arteries. The pigs were monitored weekly using an ultrasound machine (GE Medical Systems, Milwaukee, WI, USA) for up to 30 days.

### Surgical procedure

**Fig. 2:**
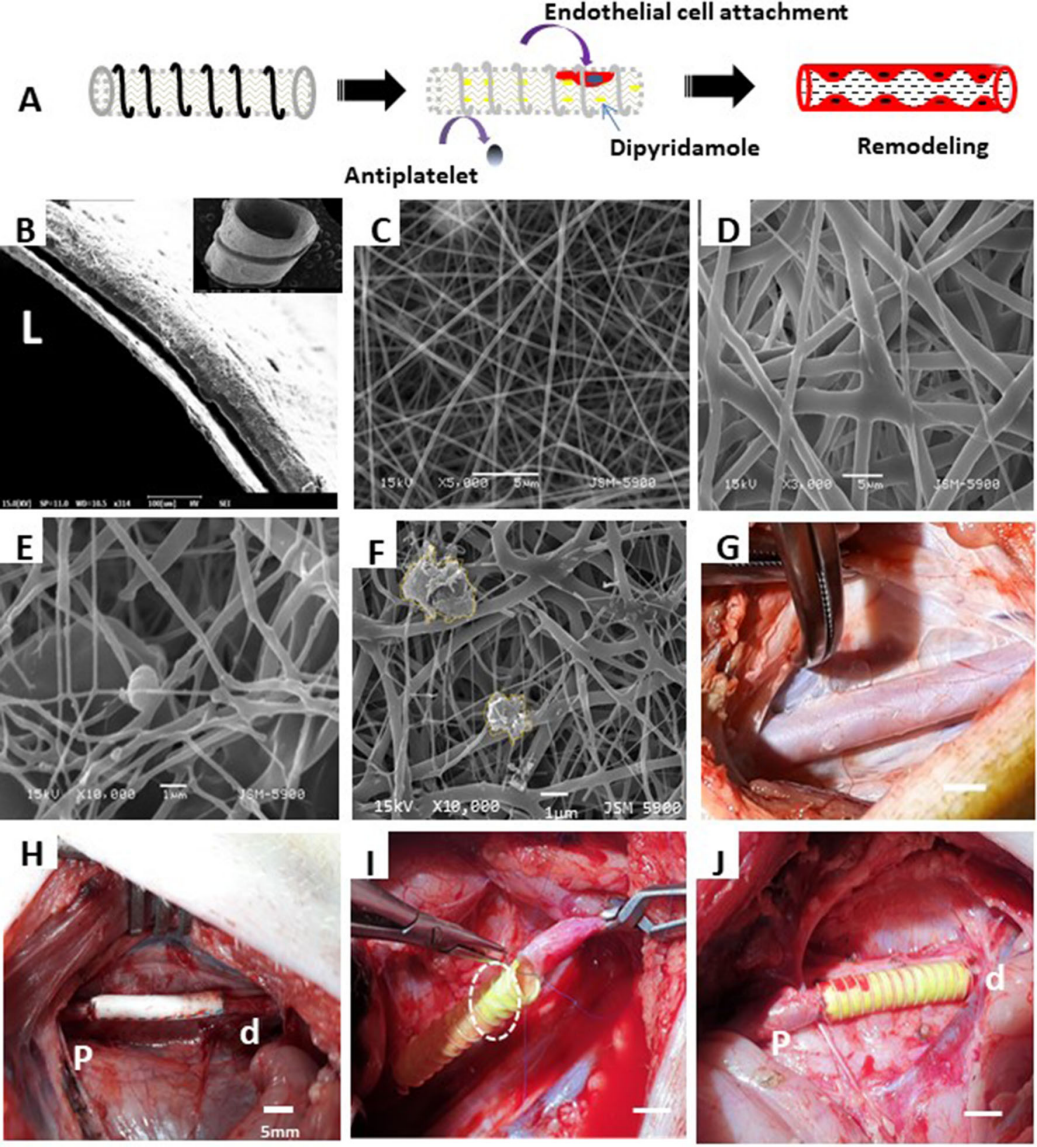
Overview of the surgical procedure and graft characterization. **A** Schematic illustration of direct acellular graft implantation and the proposed remodeling process. **B** SEM image of electrospun graft cross-section showing a double layer and fibrous topology (scale bar: 100 μm). L = lumen of the graft. Insert (side view) of the graft (scale bar: 5,000 μm). **C** The luminal surface of the graft is composed of PDO+10%DY nanofiber alone (scale bar: 5 μm). **D** The abluminal part of the graft is composed of PCL/PDO+10%DY nanofiber (PDO fiber diameter range: * 250–300 nm; PCL fiber diameter = 2 μm) (scale bar: 10 μm). **E** SEM image of the drug-loaded graft after incubation in platelet-rich plasma (scale bar: 1 μm). **F** SEM image of graft material without a drug (scale bar: 1 μm). The yellow dotted circles indicate adherent platelets with spread-like morphologies. **G** Gross image of the carotid artery exposed from adherent tissue. **H** e-PTFE graft after anastomosis. **I** Reinforcement indicated by white dotted circle on PCL/PDO+10%DY graft aided suture placement by retaining an open vessel structure. **J** PCL/PDO+10%DY graft after end-to-end anastomosis to the native vessel. Both grafts were implanted end-to-end with 8–0 non-resorbable polypropylene running stitches. P, proximal to graft; d, distal to graft (scale bar: 5 mm)

The pigs were administered 200 mg of aspirin, the day before surgery. On the day of surgery, the pigs were anesthetized by intramuscular injection of ketamine (0.25 ml kg^−1^), xylazine (3 mg kg^−1^; Rompun®; Bayer AG, Leverkusen, Germany), and azaperone (6 mg kg^−1^; Strensil®; Janssen-Cilag, Neuss, Nordrhein-Westfalen, Germany). They were then weighed and placed in the supine position, and their limbs were restrained on an operating table. The anesthesia was monitored, and when necessary, a muscle relaxant (ATRA®) was administered via the ear vein. An 8-gauge endotracheal tube was carefully inserted through the pig’s mouth to supply oxygen/anesthesia via a mechanical ventilator (Datex-Ohmeda®; GE Medical Systems), while vital parameters (e.g., respiration rate, heartbeat, and body temperature) were monitored. While isoflurane was administered (1.5–2%; Baxter Healthcare, Deerfield, IL, USA), the neck region was shaved and disinfected with povidone and alcohol. A 10-mm incision was made on either side of the neck region to expose the carotid artery. After the carotid artery had been isolated and clamped, 200 U/kg of heparin solution was injected intravenously as an anticoagulant. After 3 min, the proximal and distal ends of the common carotid artery were clamped with non-crushing clamps to stop blood flow. An approximately 1.5-cm segment of the native carotid artery was resected from the mid-section of the clamp. A 20-mm length of ethylene oxide-sterilized *e*-PTFE or PCL/PDO + 10%DY vascular graft was anastomosed to the proximal end, and then to the distal end by running stitches using 8-0 Prolene sutures (Johnson and Johnson Medical, Cincinnati, OH, USA). The proximal clamp was released to establish blood flow through the vessel, and continuous hemodynamic monitoring by pulse oximetry (for measurement of CO_2_ and temperature) was maintained throughout the procedure. Finally, the incision was closed in the standard manner with absorbable 2-0 sutures (Ethicon, Neuchatel, Switzerland) for muscle and 5-0 sutures for the skin. After confirmation of blood flow through the graft by Doppler ultrasonography, the animal was woken and transferred to its cage. Aspirin (100 mg/day) and dipyridamole (75 mg/day) were administered for 3 days after surgery. Figure 2 shows an overview of the surgical procedure and graft characterization.

### Postoperative care

The pigs were treated with analgesic (tramadol twice a day) for up to 1 week after the operation and allowed free access to food and water. Movement of the hind limbs was monitored daily for signs of spinal cord ischemia. While graft patency was monitored with an ultrasound machine weekly (weeks 0–4 post-surgery), the peak systolic velocity (PSV; proximally, within the graft, and distal to the graft) was also recorded.

### Graft description

In each group, four samples were explanted 4 weeks after surgery. Before graft harvesting, the pigs were anesthetized, and the grafts were retrieved alongside a 2-cm native vessel as an anatomical control. The retrieved grafts were gently rinsed in saline to remove residual blood. The explanted grafts were dissected in cross-section, and the patency and tissue formation on the luminal surface was observed and photographed. The grafts were further divided for histological and scanning electron microscopy (SEM) examination.

### Scanning electron microscopy

The grafts were rinsed with normal saline after explanation and subsequently fixed in 2.5% glutaraldehyde for 4 h. The samples were then transferred into PBS and allowed to stand overnight, after which they were rinsed five times with PBS. Samples were dehydrated with a series of ethanol solutions of increasing concentration (50%, 70%, 80%, 90%, and 100%, twice for 15–20 min each time). For critical point drying, we transferred the samples into a fresh solution of 1:2 hexamethylenediamine (HMDS):ethanol 100% for 20 min. The samples were transferred to a fresh solution of 2:1 HMDS: ethanol for a further 20 min. Finally, the samples were transferred into 100% HMDS for 20 min; this step was repeated, after which the samples were allowed to stand overnight in a fume hood with the cover loosely open. The samples were then sputter-coated with gold and examined by SEM (EM-30AX; COXEM Co., Ltd., Daejeon, South Korea). Similar to a previous study [38], we performed an average of 20 random measurements of each fiber sample from SEM images of 2 fiber mats using ImageJ (ImageJ, National Institute of Health, MD, USA) software to analyze the pore size of the inner and outer layer.

### Histological analysis

Cryosections (8-µm-thick) were deparaffinized by placing them in xylene three times, for 5 min each time, followed by hydration through a descending alcohol series (99.9%, 95%, and 70%) and double-distilled water (ddH_2_O). Hematoxylin and eosin (H&E), Masson’s trichrome (MT), safranin O (SO), and Verhoeff-Van Gieson (VG) staining were performed according to the manufacturer protocols. Subsequently, the samples were dehydrated through an ascending ethanol series (50%, 70%, 80%, 90%, and 100%, twice for 15–20 min each) and mounted on slides for observation under an optical microscope.

### Immunofluorescence staining

Briefly, paraffin was removed from the 8-µm-thick cryosections of explants by placing in xylene three times, for 5 min each time, followed by three different alcohol concentrations (99.9%, 95%, and 70%) and subsequent washing with ddH_2_O. The samples were then transferred to 3% hydrogen peroxide for 10 min and washed with PBS three times, for 5 min each time. Antigen retrieval was performed by incubation in citrate buffer (pH 6.0) for 10 min at 97 °C. The samples were blocked with 1% bovine serum albumin (BSA) in PBS for 1 h and washed with PBS three times, for 5 min each time. Immunohistochemical staining was performed using mouse anti-human α-smooth muscle actin (SMA) antibody (mca5781ga; Bio-Rad, Hercules, CA, USA) or anti-cardiac myosin heavy chain (MHC) monoclonal antibody (Invitrogen, Carlsbad, CA, USA) as primary antibodies. Labeling was visualized using Alexa Fluor 597-conjugated goat anti-mouse secondary antibody (Invitrogen). ECs were stained using FITC-conjugated anti-Von Willebrand factor (vWF) primary antibody (mbs2016029; MyBioSource) and Alexa Fluor 488-conjugated goat anti-rabbit secondary antibody (Invitrogen). Collagen I/III was stained using rabbit anti-pig collagen I/III (Bio-Rad) polyclonal antibody. Newly recruited macrophages were visualized using a monoclonal antibody to CD68 as the primary antibody (Invitrogen) and Alexa Fluor 597-conjugated goat anti-mouse secondary antibody (Invitrogen). The nuclei were stained by incubation in 4′,6-diamidino-2-phenylindole (DAPI) < . All antibodies were used according to the manufacturer’s protocols. The samples were washed with PBS three times for 5 min each time and mounted onto slides using a medium. Fluorescence images were captured using a laser scanning confocal microscope; ZEISS LSM 800. Native pig carotid artery was used as a positive control.

## Results

### General examination

Two groups of five pigs each were implanted with either PCL/PDO + 10%DY or *e*-PTFE grafts in the left and right carotid arteries. All pigs were monitored at four experimental time points (1, 2, 3, and 4 weeks after implantation) by ultrasonography. All pigs survived for 4 weeks. Surgery duration and vessel clamp time were similar between the two grafts, as summarized in Table 1. After implantation, the PCL/PDO + 10%DY grafts pulsated in synchrony with the blood flow. No plasma leakage was seen in the PCL/PDO + 10%DY grafts, but obvious plasma leakage was seen in several *e*-PTFE grafts (Supplementary Video 1).

**Table 1:** Surgical parameters in the two study groups

Graft	*e*-PTFE	PCL/PDO
Number of samples	5	5
Pig weight (kg)	25–35	
Anastomosis time (min)	60 ± 8	70 ± 10
Vessel clamp duration (min)	75 ± 10	85 ± 8
PC_O2_ (mmHg)	37 ± 5	
BP before the procedure (mmHg)	138/93–119/78	
BP after the procedure (mmHg)	110/78–112/76	

### Electrospun drug-loaded graft inhibited platelet activation

Figure 2A illustrates the proposed remodeling process of fabricated PCL/PDO + 10%DY graft. Similar to our previous design, i.e., with slight modifications [36], the graft described here consisted of double layers of drug-loaded nano/macro fibers with a porous network (Fig. 2B–D). The luminal layer was made from thin (~ 250–300 nm), rapidly degradable electrospun PDO nanofibers (Fig. 2C) intended to prevent plasma leakage, as the loaded drug inhibits clot formation on the surface. The abluminal layer consisted of a combination of PCL (~ 2 µm) and PDO fibers, spun together to enhance the strength of the graft. The overall thickness of the graft was ~ 250–300 µm (Fig. 2B) with ~ 100% interconnected pores (Fig. 2C, D), necessary for cell colonization and rapid host integration. When the pore size of PDO and PCL/PDO layer was determined using ImageJ, the PDO layer had low pore size than PCL/PDO (5.5 ± 1.5 µm against 24 ± 5 µm). this might be due to larger pores introduced by PCL microfibers. We used 3D printing to attach a PCL reinforcement (160 µm) around the graft, to enhance strength and prevent aneurysmal dilation as the nanofibers degrades *in vivo*. The grafts were stored at room temperature after sterilization with ethylene oxide. The inner diameter of the graft (5 mm) could be measured easily using a Vernier caliper or ruler (Fig. 1). The release of antithrombotic drugs from the nanofiber matrix prevented platelet activation (Fig. 2E–F). Platelets adhering to the drug-loaded graft show a rounded (i.e., not activated) shape (Fig. 2E), while those on grafts without the drug were spread-like (i.e., activated) morphology (Fig. 2F). Either the PCL/PDO + 10%DY or *e*-PTFE graft was implanted after exposure to the native carotid artery (Fig. 2G). The grafts were readily distinguishable from the native artery after implantation (Fig. 2H–J). The reinforcement on the PCL/PDO + 10%DY graft made the anastomosis procedure easier, as it helped to maintain the cylindrical shape (Fig. 2I).

### Drug release profile

**Fig. 3:**
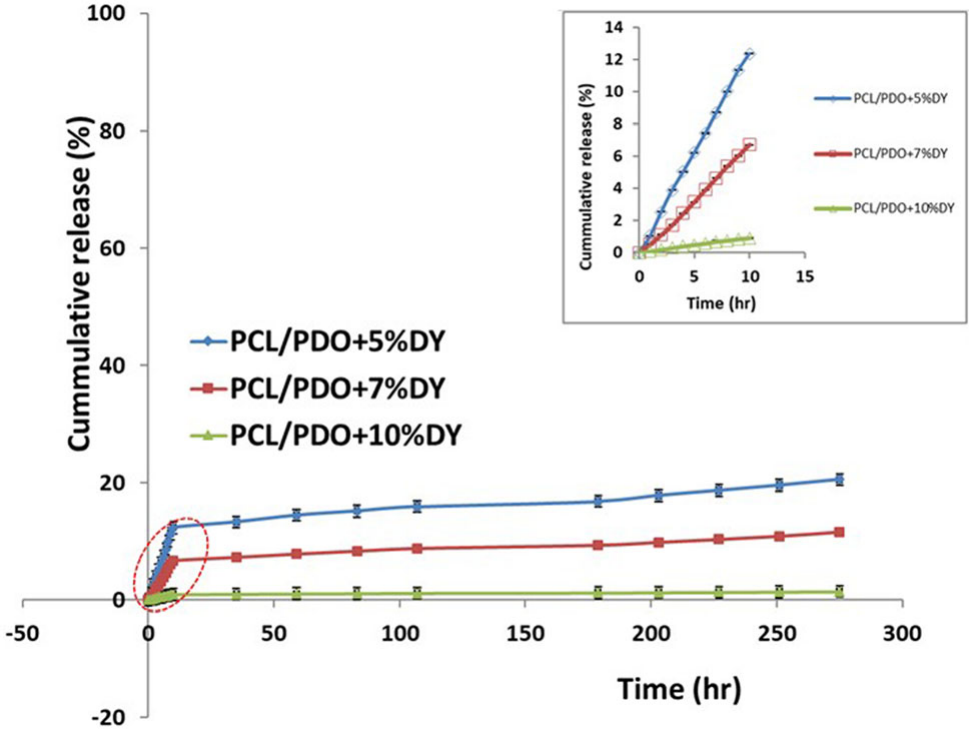
*In vitro* cumulative drug release of dipyridamole from nanofibers containing various concentrations of drug (PCL/PDO + 5%DY, PCL/PDO + 7%DY, and PCL/PDO + 10%DY). The insert shows a higher magnification view of the area indicated with a red dotted circle

In this experiment, we compared the release kinetics of dipyridamole at three concentrations in the graft material over 35 h (Fig. 3). The cumulative release of the samples reached a plateau after 10 h, following a slow release over the experimental window (275 h). As expected, a slower release was observed for PCL/PDO + 10%DY compared to the PCL/PDO + 5%DY scaffold over the whole release process. At 10 h 12.3%, 6.7%, and 0.9% of the drug were released from PCL/PDO + 5%DY, PCL/PDO + 7%DY, and PCL/PDO + 10%DY drug-loaded scaffolds, respectively. Sustained release of drug from the PCL/PDO + 10%DY drug-loaded scaffold could be suitable for inhibiting platelet activation on the implanted graft surface over a long period, thus we choose this sample for the *in-vivo* experiment.

### Graft patency/synchronous pulsation with host artery

**Fig. 4:**
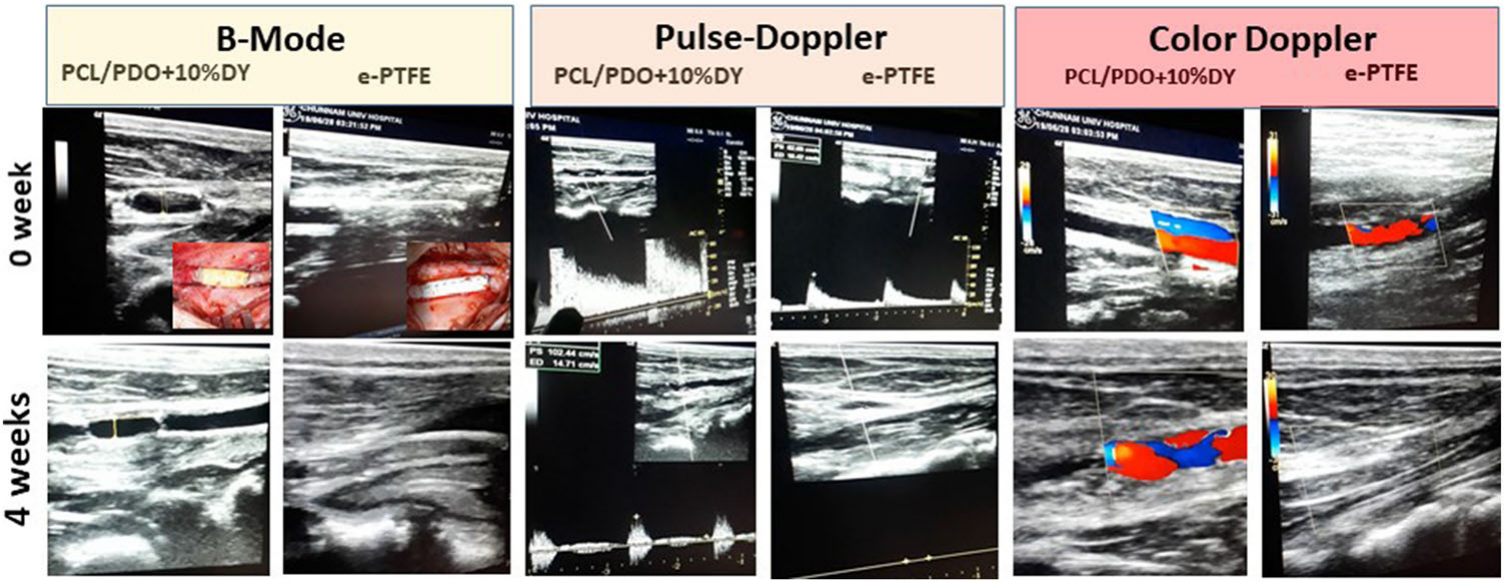
Noninvasive serial ultrasonographic monitoring of grafts implanted in a pig carotid artery interposition model. Vertical panels 1 and 2 from left show B-mode ultrasonograms of PCL/PDO + 10%DY and *e*-PTFE grafts at 0–4 weeks (inset shows a gross image obtained during post-surgical anastomosis of both grafts). Panels 3 and 4 show the serial pulse-wave Doppler ultrasonographic examination results, while panels 5 and 6 show color Doppler duplex ultrasound images. Color Doppler indicates there was no flow through *e*-PTFE graft after 1 week indicating occlusion

Graft patency was monitored using a combination of ultrasonography and manual palpation of the carotid artery pulsation, as shown in Fig. 4. After surgery, all experimental animals were palpated to confirm pulsation of the graft. The pigs were closely monitored daily, for signs of mental distress, food intake, hind limb function, and physical activity. The images are shown on the first row in Fig. 4 (implantation day 0) indicate that all grafts were patent. However, pulse-wave and color Doppler ultrasound at weeks 1–4 of implantation indicated significant differences in patency between PCL/PDO + 10%DY and *e*-PTFE grafts as early as 1 week after the operation (some data not shown). At the end of the follow-up period (4 weeks), Doppler ultrasound revealed that, for the PCL/PDO + 10%DY grafts, patency of up to 96% (4/4) was maintained, in contrast to 0% (0/4) for *e*-PTFE grafts. PCL/PDO + 10%DY grafts showed excellent patency and strong synchronous pulsation with the host artery (Supplementary Video 2), and there was no evidence of stenosis or aneurysm. The regular and clear pulsation seen at 4 weeks indicated excellent integration with the host tissue. Before occlusions were observed in the *e*-PTFE grafts, retrograde flow signals were often seen at the distal arteries, suggesting thrombus formation.

### Ultrasound monitoring

**Fig. 5:**
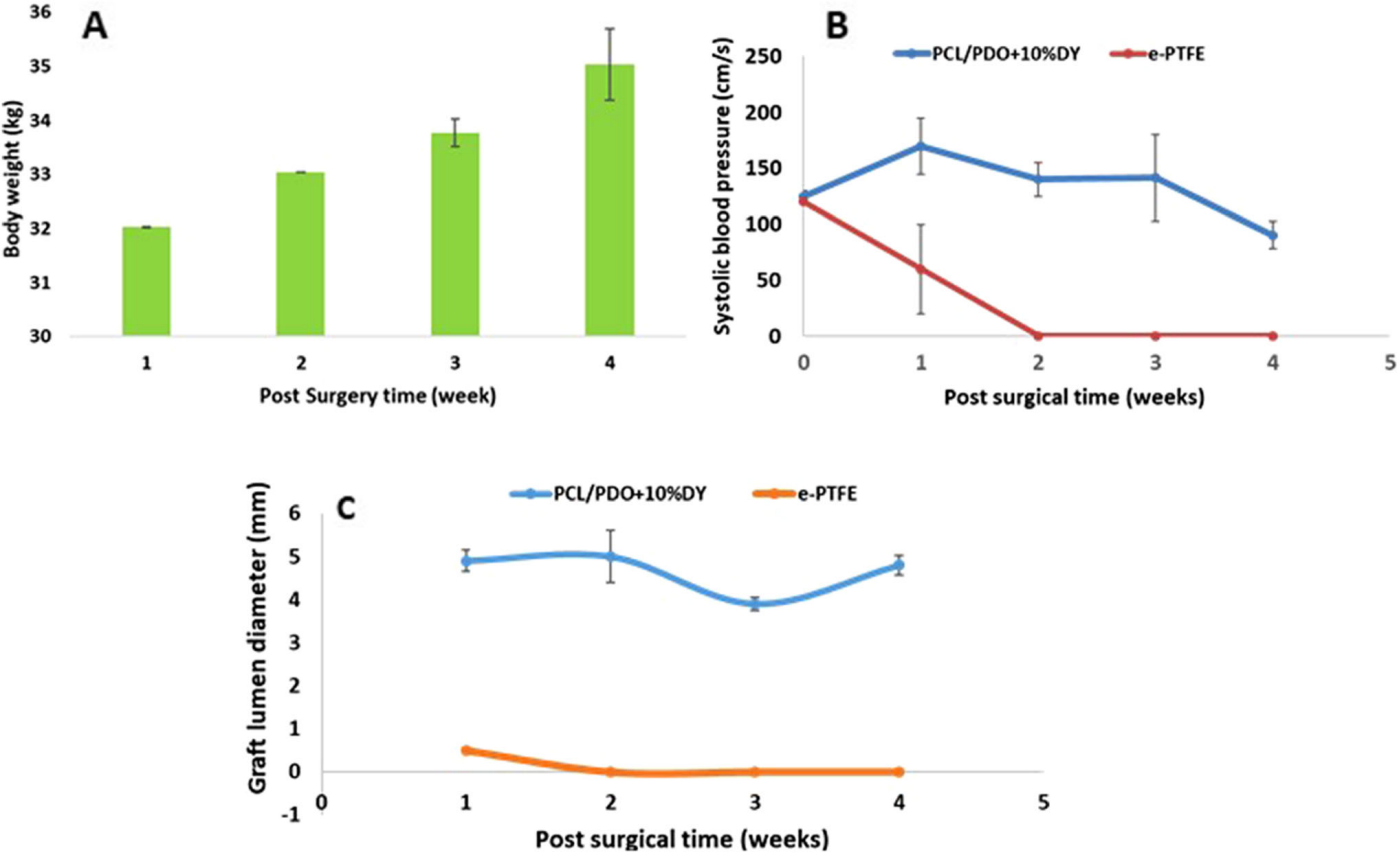
**A** Variation in animal body weight after surgery **B** Ultrasound measurements of graft mid-luminal diameter, and **C** Systolic blood flow through the PCL/PDO + 10%DY and *e*-PTFE grafts 1–4 weeks post-surgical implantation

All animals showed healthy weight gain 4 weeks after surgery, with an average weight increase of 9.8% throughout the study period (Fig. 5A). Ultrasound measurements of the PCL/PDO + 10%DY mid-graft diameter (Fig. 5B) showed a decreased at 3 weeks and then increased at 4 weeks (by 14.29%), while *e*-PTFE grafts were all occluded from 2 weeks after surgery. The peak systolic blood flow through the PCL/PDO + 10%DY grafts fluctuated over the study period, but the values were within the normal physiological range (Fig. 5C). Furthermore, there were no increases or decreases in downstream blood flow velocity at the distal end, indicating no pressure gradient within the graft.

### Graft outcomes

**Fig. 6:**
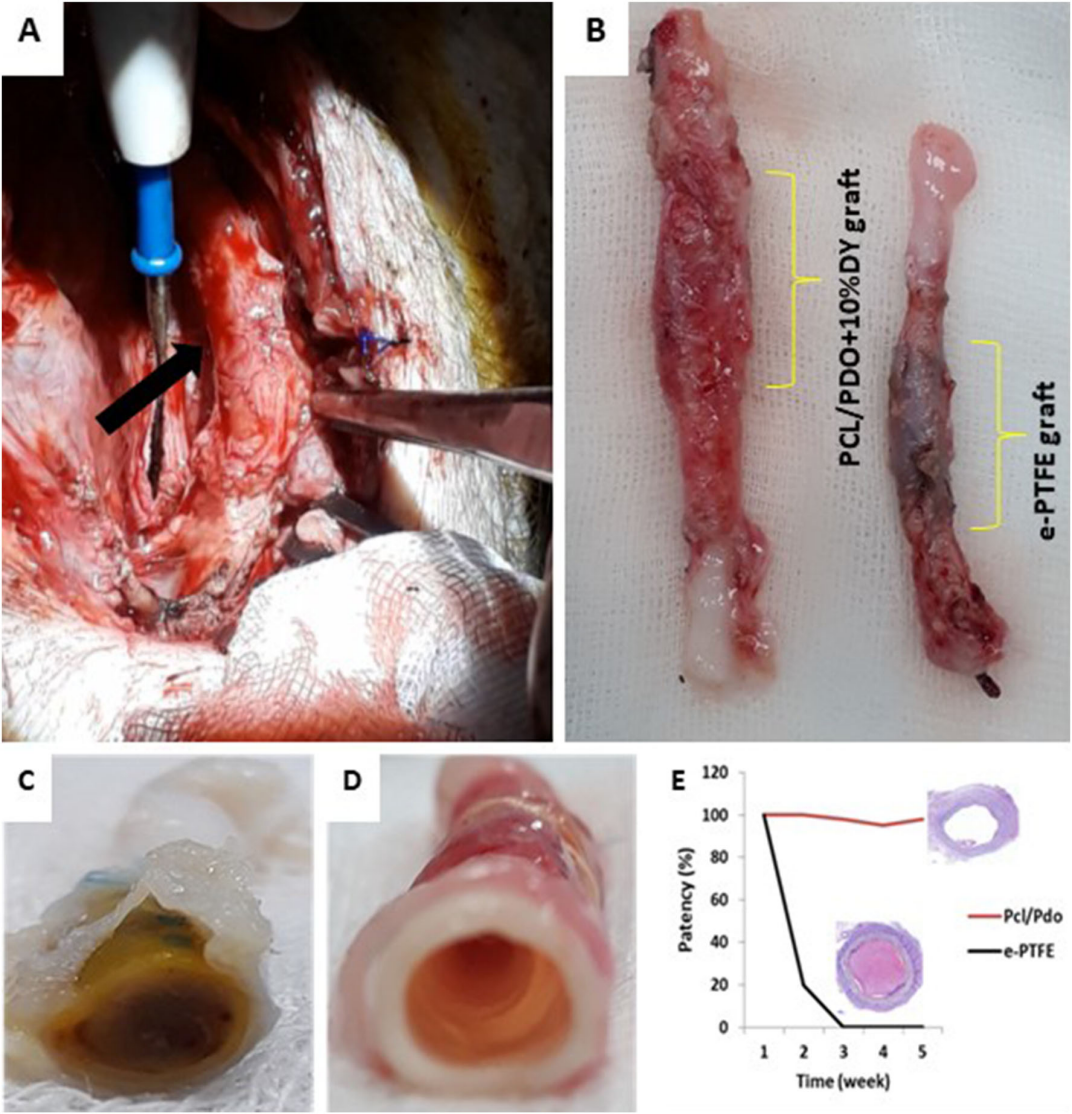
Gross images of explanted grafts after 4 weeks. **A** Surgical view of PCL/PDO + 10%DY grafts in situ (black arrow). The head is in the lower part of the image. **B** Explanted PCL/PDO + 10%DY and *e*-PTFE grafts with portions of the native carotid artery in both ends. **C** Gross image showing a thrombosed *e*-PTFE graft; the PCL/PDO + 10%DY graft was patent. **D** Patency checks of PCL/PDO + 10%DY and *e*-PTFE grafts over 4 weeks. **E** Patency evaluation of grafts over 4 weeks

On gross examination (Fig. 6), no signs of infection were observed in the graft region in either group (Fig. 6A), and the other organs appeared normal. Interestingly, the explanted PCL/PDO + 10%DY grafts were almost indistinguishable from the native part of the vessel because of the high degree of remodeling with abundant capillary vessel formation. All retrieved PCL/PDO + 10%DY grafts were patent and retained their tubular shape. However, the lumen of *e*-PTFE grafts was occluded by thrombi (Fig. 6C, D). The anastomoses at both ends of the grafts were intact and no pseudoaneurysms were detected. Graft patency was assessed by weekly ultrasonography and finally confirmed by histopathological examination (Fig. 6E). All *e*-PTFE grafts were occluded at 2 weeks of implantation, while PCL/PDO + 10%DY grafts remained patent at the end of the study.

### Histological examination

**Fig. 7:**
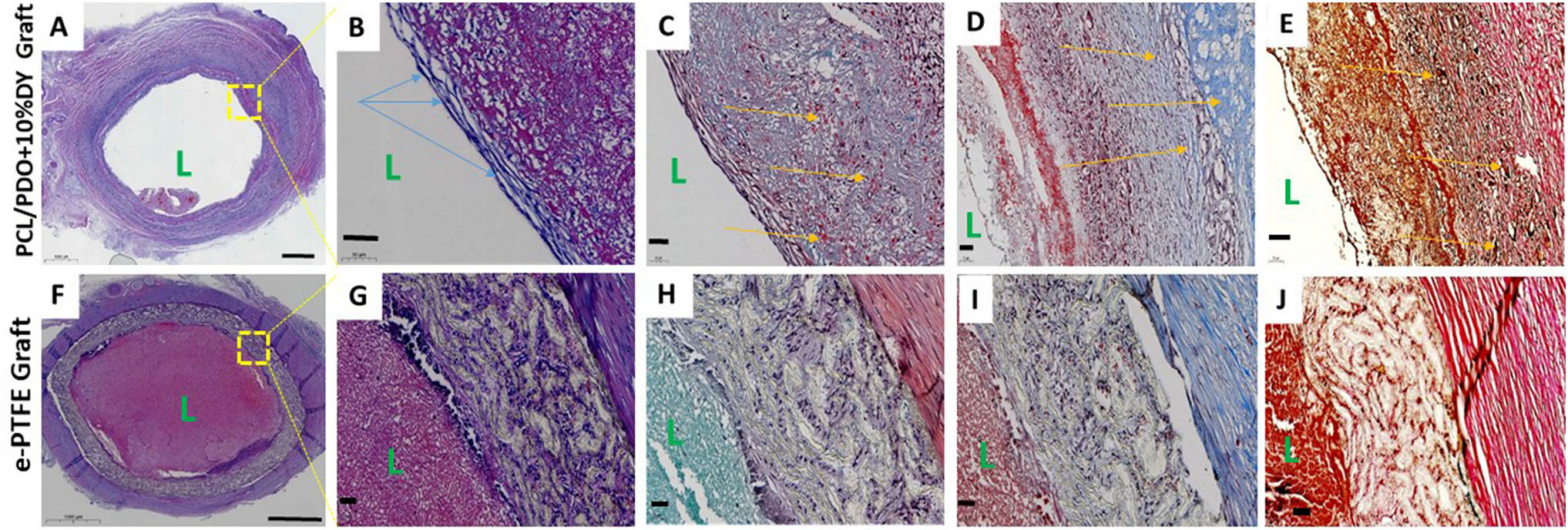
**A**–**J** Histological images of explanted grafts at 4 weeks after surgery (top panel: cross-section of a PCL/PDO + 10%DY graft; bottom panel: the corresponding e-PTFE graft (scale bar = 1000 µm)). **B** H&E staining of the portion of (**A**) with the blue arrow showing confluent ECs covering the luminal surface (scale bar: 50 µm). L, luminal area. **C** Safranin O staining to identify deposition of GAGs (stained red) (scale bar: 50 µm). **D** Masson’s trichrome staining shows early deposition of collagen (yellow arrow) (scale bar = 50 µm). **E** Verhoeff’s staining indicated elastin deposition (in black) (scale bar = 50 µm). **F**–**J** The bottom row shows corresponding stained images of the *e*-PTFE graft. Middle part = thrombus

Figure 7 shows histological images of PCL/PDO + 10%DY and *e*-PTFE grafts 4 weeks after the implantation. Repopulation of the graft by host cells via the porous microstructure of PCL/PDO + 10%DY indicated that little remodeling had taken place. The thin layer of spindle-shaped cells on the luminal surface of the PCL/PDO + 10%DY graft indicated EC coverage (Fig. 7B). A significant quantity of extracellular matrix (ECM) components on the graft indicated the marked impact on the host cells. The red arrow indicates a significant amount of glycosaminoglycans in the graft (Fig. 7C). ECM consisting mostly of collagen and elastin was observed in the graft, along with more organized medial and neoadventitial layers (Fig. 7D, E).

### Immunofluorescence staining

**Fig. 8:**
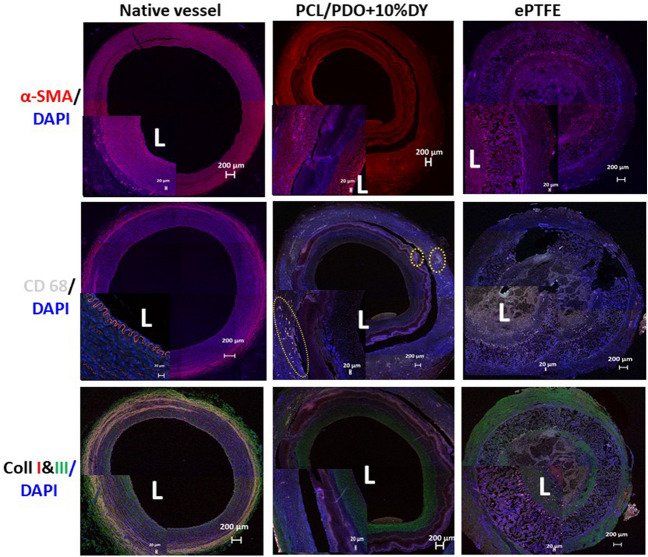
Remodeling of grafts at 4 weeks after implantation. Immunofluorescence staining: The top row shows positive staining of smooth muscle cells (red) counterstained with DAPI (blue) at 4 weeks (scale bar: 500 µm). The inset (scale bar: 200 µm) showing PCL/PDO + 10%DY indicates that the wall of the graft was well infiltrated by smooth muscle cells. The *e*-PTFE graft at 4 weeks showed a low level of smooth muscle cell infiltration, as revealed by the higher magnification view in the inset image. L = lumen of the graft. The middle row shows positive staining (bright red) of cells on the PCL/PDO + 10%DY graft for CD68 (cluster demarcated by broken yellow lines). Such cells were not observed on the *e*-PTFE graft, as most of the graft was occluded after 1 week after implantation. The lower row shows positive staining of the PCL/PDO + 10%DY graft for collagen I (red) and collagen III (green).

Vascular smooth muscle cells (VSMCs) are essential for good vessel function. Our immunofluorescent staining data at 4 weeks indicated the presence of smooth muscle cells and a more organized medial layer for the PCL/PDO + 10%DY graft (Fig. 8). Macrophages, which are cells that actively participate in vascular graft remodeling via immune-modulation, were observed in the graft interstices. However, the prolonged presence of these special cells according to previous report could trigger a chronic inflammatory response [39]. Newly recruited CD68^+^ cells (pan-macrophage marker) were positively stained, as shown by the yellow circles distributed throughout the graft, especially within the vicinity of polymer remnants (Fig. 8).

The amount of ECM deposition on the graft indicates the impact of host cell infiltration. The graft wall contained significant amounts of collagens I and III (Fig. 8), and the clear circumferential alignment of these ECM proteins resembles the native vasculature. In contrast to the native artery, PCL/PDO + 10%DY grafts were less compact and more cellular. The large amounts of collagens I and III in the tunica medial and external layer provided good mechanical support to the vessel, in terms of strength and flexibility, as the nanofibers disappears.

**Fig. 9:**
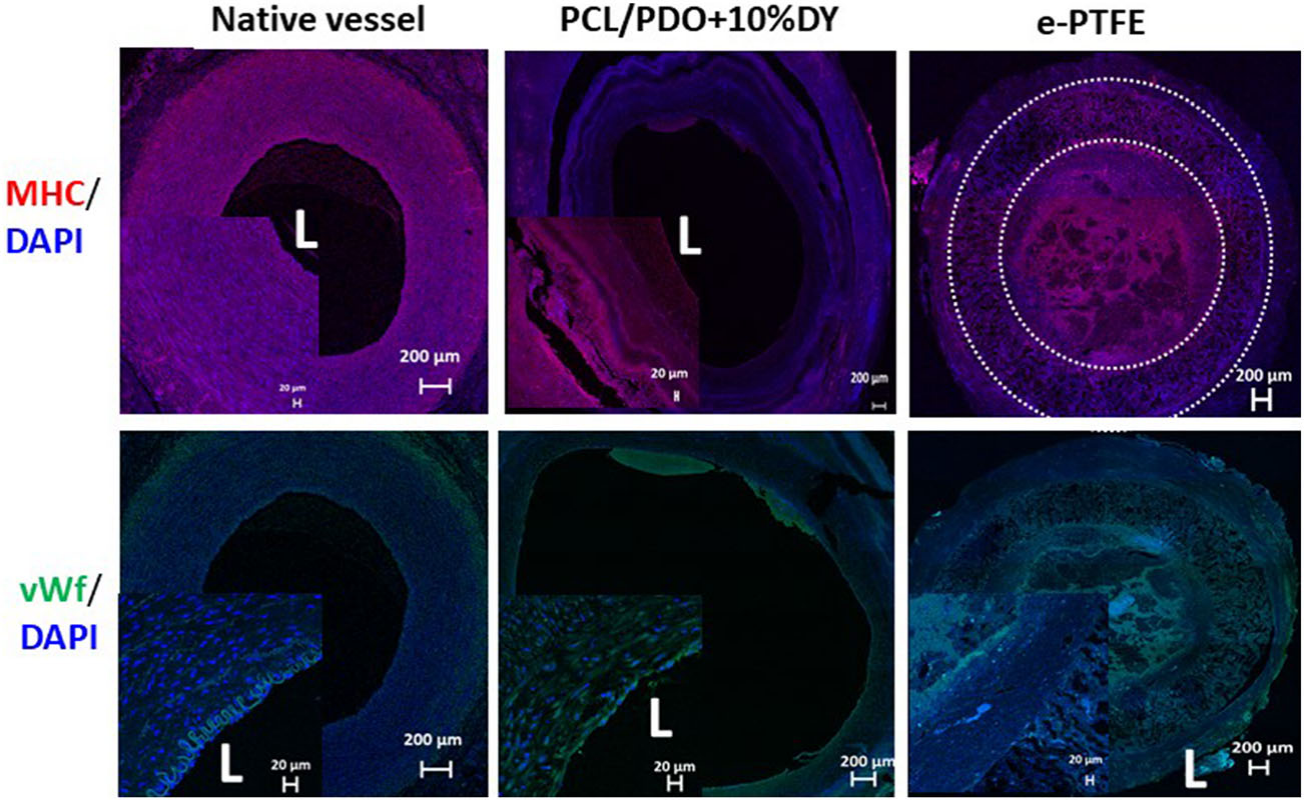
Reendothelialization of the grafts at 4 weeks. The top row shows the distribution of contractile smooth muscle cells (MHC, red) and nuclei counterstained with DAPI. The bottom row shows representative micrographs of immunohistochemical staining for vWF (green) on cross-sections of the native carotid artery, PCL/ PDO+10%DY graft, and e-PTFE graft at 4 weeks. The ECs on the luminal surface/graft wall were stained green. Nuclei were counterstained with DAPI (blue). scale bar: 200 μm. L = lumen of the graft and a thin white dotted circle was used to indicate *e*-PTFE graft. The image clearly showed a thin layer of endothelial cells present on the PCL/PDO+10%DY graft (inset scale bar: 20 μm)

Positive staining for MHC in the neoartery indicated a contractile smooth muscle phenotype (Fig. 9). This motor protein is a late-stage marker of the differentiation of smooth muscle cells. There have been few reports of positive staining for MHC in vascular grafts [40, 41]. Immunohistochemistry of the graft cross-section with vWF was carried out to confirm the presence of ECs, as described in previous reports [8, 42, 43]. At 4 weeks, the PCL/PDO + 10%DY graft cross-section showed positive staining for ECs covering the luminal surface (Fig. 9), and this confirmed our H&E staining results.

### Endothelization of the graft after 4 weeks

**Fig. 10:**
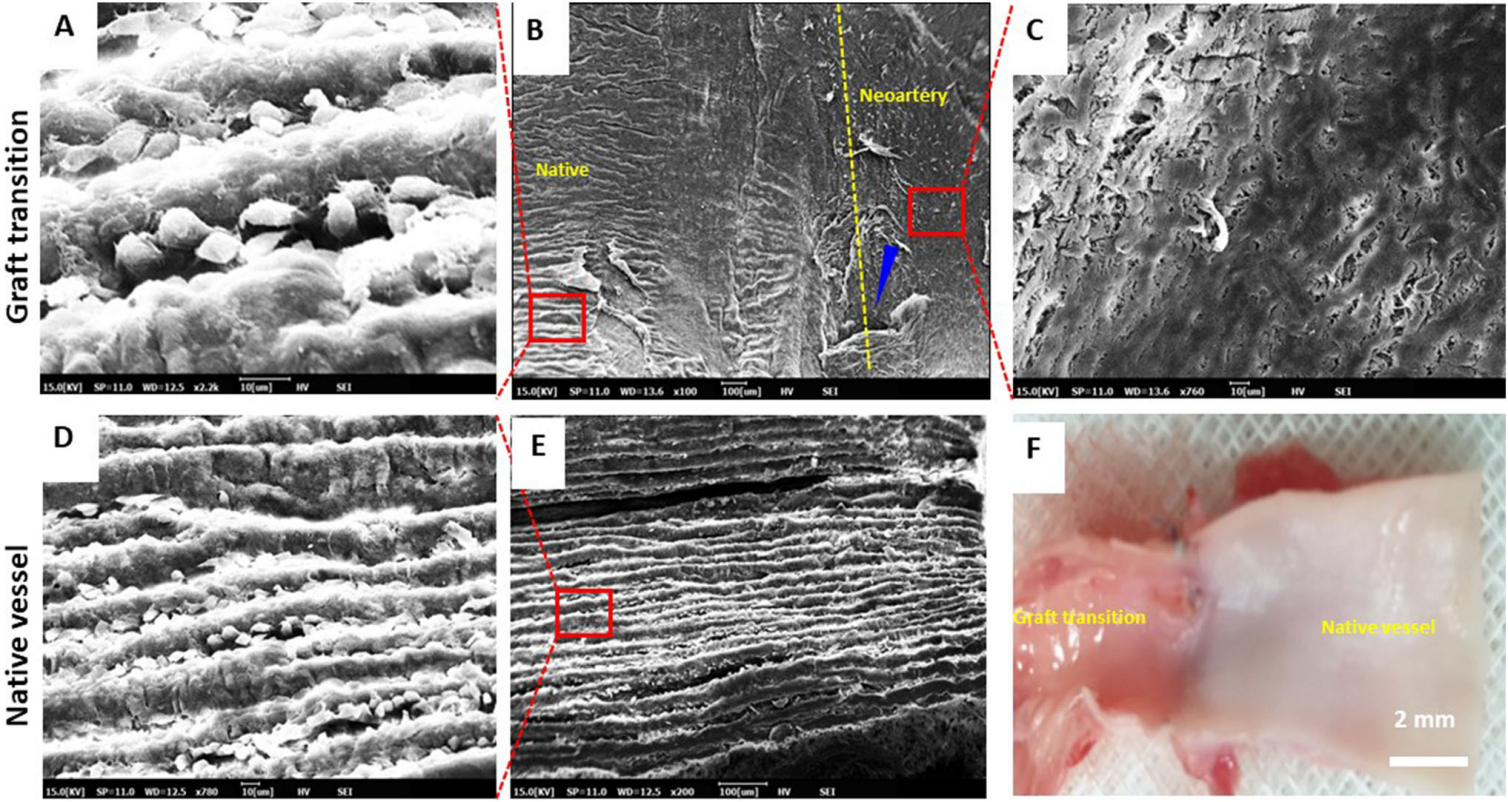
Re-endothelialization of the neoartery at 4 weeks. **A** SEM image of the native vessel distal to the graft. **B** Explanted graft with 1 mm part of the native vessel. The blue arrow indicates the suture hole, while the yellow broken line indicates the anastomosis site. **C** ECs completely covered the luminal surface of the graft. **D** Higher magnification image of a typical autologous vessel -luminal surface with folded morphology and ECs in the interstices. **E** A typical surface of the native vessel lumen. **F** Gross macroscopic image of an explanted graft harvested with a small part of the native vessel. The optical image shows there was an obvious difference in ECM content between the neoartery and native vessel. Scale bar: 2 mm

ECs are derived from the differentiation of circulating autologous ECs or endothelial progenitor cells [44, 45]. Here, we compared SEM images of the luminal surfaces of both the explanted PCL/PDO + 10%DY grafts and native autologous vessels. The native part of the vessel showed typical microvilli or Weibel–Palade bodies, which are membrane-enclosed rod-shaped organelles found in ECs (Fig. 10A). This structure is selectively permeable for certain chemicals and promotes the movement of white blood cells from blood to tissue, and the transport of waste and CO_2_ from tissue to blood. However, PCL/PDO + 10%DY graft surfaces were smooth and clean, with ECs fully covering the luminal surface (Figs. 9 and 10B, C). Neither thrombi nor platelet aggregation was observed on the surface. The typical luminal surface of the native carotid artery is shown in Fig. 10D, E. The PCL/PDO + 10%DY graft showed the absence of this folded morphology at 4 weeks due to the early stages of endothelium regeneration. More so, optical observation of the explant confirmed slight difference in ECM content (Fig. 10F). Rapid adhesion and proliferation of ECs are essential for preventing thrombosis, and long-term graft survival [46]. These results are consistent with the findings of immunofluorescence and histological analyses.

## Discussion

In our previous study, we explored the short-term (2 weeks) performance of a new reinforced antithrombotic acellular graft in a porcine carotid artery model [36]. In that study, we investigated the mechanical and biocompatibility of the graft both *in vitro* and *in vivo* which showed promising for use as a vascular alternative. There is accumulating evidence that small-diameter grafts should be tested in vessels with low blood flow, such as the carotid artery, rather than high-flow vessels such as the aorta. The carotid artery undergoes more frequent twisting and stretching due to cervical mobility [47]. Therefore, vascular grafts can be more rigorously tested for patency, mechanical flexibility, and muscular remodeling in the carotid artery. In the present study, we used 4 weeks as the terminal time point and compared the performance of the PCL/PDO + 10%DY graft with commercially available *e*-PTFE grafts in a porcine carotid artery model. We analyzed the patency, drug release, morphological changes, ECM production, and cellular infiltration.

Intimal hyperplasia and thrombosis remain the greatest challenge in vascular surgery, especially in small-diameter vessels. Despite numerous investigations, graft failure due to intimal hyperplasia and thrombosis has yet to be eliminated. Furthermore, for clinical translation, synthetic vascular grafts require certain characteristics, such as cost-effectiveness, cell-free status, good mechanical properties, biodegradability, a highly porous structure for cell infiltration, easily scalable fabrication, and off-the-shelf availability. Vascular substitutes should also be anti-thrombogenic, have the structure of VSMCs/ECs [48], and be capable of regenerating into new functional tissue [49, 50].

The most interesting findings of the present study regarding small-diameter (< 6 mm) PCL/PDO + 10%DY grafts at 4 weeks are that the grafts were patent and showed synchronous pulsation with the native vessel, whereas all *e*-PTFE grafts failed due to thrombosis at 2 weeks, similar to previous reports [51, 52]. Additionally, though there was no significant difference in clamping duration for both grafts, however, most *e*-PTFE grafts show remarkable plasma leakage after anastomosis which was not observed on PCL/PDO + 10%%DY grafts. Previous studies have explored the potential of PCL and PDO to fabricate vascular grafts [53]. Although PCL has good biocompatibility, its long-term presence when used alone to fabricate vascular grafts can result in calcification or failure [53, 54]. We departed from the traditional design strategy of either using slowly degradable polymers, such as PCL, which may result in calcification in the long term, or rapidly degradable polymer, which may result in aneurysm dilatation due to slow tissue regeneration.

Here, we used an electrospinning approach to combine a rapidly degradable polymer (PDO) as the luminal layer and a combination of PCL and PDO, spun simultaneously, as the abluminal layer. employing this strategy, the luminal layer will be replaced with endothelium as it degrades faster, while the thin abluminal layer will degrade slowly over time. The polymer solutions were all doped with an antithrombotic drug (dipyridamole) intended to inhibit clot formation. Finally, the graft was reinforced by 3D printing, using biodegradable PCL to increase the mechanical strength for ease of surgical handling. After 4 weeks *in vivo*, the graph showed a high patency rate with complete endothelial coverage and no thrombi on the luminal surface, coupled with the formation of medial and neoadventitial layers; in contrast, the *e*-PTFE graft occluded at 2 weeks. A comprehensive assessment of remodeling cell types, such as inflammatory cells, and ECM production further confirmed positive remodeling of PCL/PDO + 10%DY grafts during the observation period in this study.

Rapid reendothelialization is known to have antithrombogenic effects [55, 56]. Hence, both our immunohistochemical (vWf) and histological (H&E) analyses confirmed the presence of ECs at 4 weeks forming a complete monolayer. By H&E staining, we confirmed that more cells migrated into the PCL/PDO + 10%DY grafts, which may have been due to both circulating progenitor cells and transmural ingrowth of pericytes. However, e-PTFE grafts show lower host cell infiltration according to H&E staining. MT and Verhoeff’s staining also confirmed the deposition of collagen and elastin within the graft. Sufficient accumulation of these ECM proteins can act as a framework and strengthen the graft against high systemic pressure, as the polymer is gradually absorbed. Therefore, the graft degradation profile should be tailored in such a way that the degradation rate matches that of tissue regeneration. Rapid polymer degradation, i.e., before the tissue is organized, can result in rupture and aneurysmal dilation.

The results of ultrasonography demonstrated a slight reduction in diameter of the PCL/PDO + 10%DY grafts which subsequently stabilized at 4 weeks. The reduction of lumen size was apparently due to tissue formation on the luminal and abluminal sides, as confirmed by H&E staining. During this period, normal blood flow (within the physiological range) was observed. However, at 2 weeks, all e-PTFE grafts were occluded. The *in vivo* disparity between PCL/PDO + 10%DY and *e*-PTFE grafts may have been due to differences in the physical and mechanical properties of the grafts, such as the reinforcement that enhanced kink resistance in PCL/PDO + 10%DY grafts, and to the compliance of the grafts, which can significantly alter the hemodynamic flow [57].

This is the first preclinical study of *in vivo* implantation of reinforced electrospun biodegradable grafts showing synchronous pulsation with the native vessel as early as 4 weeks after implantation in a porcine model. This device is novel in that it combines nanofibers using electrospinning and 3D printing to generate an arterial substitute with compliance similar to that of the native vessel.

In conclusion, we compared the *in vivo* performance of electrospun PCL/PDO + 10%DY graft and commercially available *e*-PTFE grafts at 4 weeks in a porcine model. The ability of the PCL/PDO + 10%DY graft to promote the attachment/growth of ECs and inhibit clot formation within the experimental window supports the use of this device as a vascular substitute. Histological analysis showed that the PCL/PDO + 10%DY graft was well tolerated, and no adverse tissue reactions were observed. Furthermore, the favorable production of VSMCs and ECM proteins (elastin, collagen, and glycosaminoglycans), coupled with synchronous pulsation with the native vessel, suggested that this device has potential for clinical use. These positive results of our short-term study in a large animal model indicate the potential for future clinical application, especially in patients with cardiovascular disease.

## Supplementary Information

Below is the link to the electronic supplementary material.

Supplementary file1 (MP4 5837 kb)

Supplementary file2 (MP4 27910 kb)
